# Body composition and physical fitness in women with bulimia nervosa or binge‐eating disorder

**DOI:** 10.1002/eat.22841

**Published:** 2018-02-23

**Authors:** Therese Fostervold Mathisen, Jan H. Rosenvinge, Oddgeir Friborg, Gunn Pettersen, Trine Stensrud, Bjørge Herman Hansen, Karoline E. Underhaug, Elisabeth Teinung, KariAnne Vrabel, Mette Svendsen, Solfrid Bratland‐Sanda, Jorunn Sundgot‐Borgen

**Affiliations:** ^1^ Department of Sports Medicine Norwegian School of Sport Sciences Sognsvegen 220 N‐0806 Oslo Norway; ^2^ Department of Psychology, Faculty of Health Sciences UiT—The Arctic University of Norway Tromsø Norway; ^3^ Department of Health and Caring Sciences Faculty of Health Sciences, UiT—The Arctic University of Norway Tromsø Norway; ^4^ Research Institute of Modum Bad Vikersund Norway; ^5^ Department of Endocrinology, Obesity and Preventive Medicine Oslo University Hospital Oslo Norway; ^6^ Department of Outdoor Studies, Sports and Physical Education University College of Southeast Norway Notodden Norway

**Keywords:** binge‐eating disorder, bulimia nervosa, cardiorespiratory fitness, eating disorders, muscle strength, physical activity, physical fitness

## Abstract

**Objective:**

Knowledge about physical fitness in women with bulimia nervosa (BN) or binge‐eating disorder (BED) is sparse. Previous studies have measured physical activity largely through self‐report, and physical fitness variables are mainly restricted to body mass index (BMI) and bone mineral density. We expanded the current knowledge in these groups by including a wider range of physical fitness indicators and objective measures of physical activity, assessed the influence of a history of anorexia nervosa (AN), and evaluated predictive variables for physical fitness.

**Method:**

Physical activity, blood pressure, cardiorespiratory fitness (CRF), muscle strength, body composition, and bone mineral density were measured in 156 women with BN or BED, with mean (*SD*) age 28.4 years (5.7) and BMI 25.3 (4.8) kg m^−2^.

**Results:**

Level of physical activity was higher than normative levels, still <50% met the official physical activity recommendation. Fitness in women with BN were on an average comparable with recommendations or normative levels, while women with BED had lower CRF and higher BMI, VAT, and body fat percentage. We found 10–12% with masked obesity. A history of AN did not predict current physical fitness, still values for current body composition were lower when comparing those with history of AN to those with no such history.

**Discussion:**

Overall, participants with BN or BED displayed adequate physical fitness; however, a high number had unfavorable CRF and body composition. This finding calls for inclusion of physical fitness in routine clinical examinations and guided physical activity and dietary recommendations in the treatment of BN and BED.

## 1 INTRODUCTION

Lifestyle behaviors like diet and exercising may affect physical fitness in many ways and cause profound changes, ultimately affecting total morbidity and mortality (Mendis, 2014; Myers et al., 2015; Ross, et al., 2016). Besides body mass index (BMI) and bone mineral density, information on physical fitness (i.e., physical activity level, blood pressure, cardiorespiratory fitness [CRF], muscle strength, total body composition, and body fat distribution) in individuals with binge‐eating disorder (BED) or bulimia nervosa (BN) is scant. As such, physical fitness is rarely considered in treatment, leaving this population at an increased risk for physical comorbidity.

Individuals with BED have been described as sedentary (Hrabosky, White, Masheb, & Grilo, 2007; Vancampfort et al., 2015), whereas the opposite has been the case for those with BN. In patients with BN, the level of physical activity may even be higher than in the general population (Bratland‐Sanda et al., 2010a; Davis et al., 1997), and in about 20–30% of female patients, physical activity may be highly excessive and compulsive in nature (Dalle Grave, Calugi, & Marchesini, 2008; Davis et al., 1997; Haskell et al., 2007; Shroff et al., 2006).

CRF is closely related to physical activity and suggested as one of the most important indicators of physical fitness and mortality (Myers et al., 2015; Ross et al., 2016). The few studies that have examined CRF among women with BN (Bratland‐Sanda, et al. 2010b; Sundgot‐Borgen, Rosenvinge, Bahr, & Schneider, 2002) indicate both inferior and comparable fitness relative to normative values (Edvardsen, Hansen, Holme, Dyrstad, & Anderssen, 2013). CRF has not previously been reported for individuals with BED. Furthermore, in the general population, muscular strength has been positively correlated with self‐reported health (Payne, Gledhill, Katzmarzyk, Jamnik, & Ferguson, 2000), optimal body composition, and metabolic regulation (Artero et al., 2012; Bakker et al., 2017). Little is known about muscular strength in women with BN or BED, apart from one study of female inpatients with longstanding BN (Bratland‐Sanda et al., 2010b), which reported levels of muscular strength comparable with healthy controls.

Normal BMI values have been found for BN (Hudson, Hiripi, Pope, & Kessler, 2007; Probst et al., 2004), and overweight and obesity have been associated with BED (Bulik & Reichborn‐Kjennerud, 2003; Vancampfort et al., 2013; Wilfley, Wilson, & Agras, 2003). However, an increase in lifetime obesity within all eating disorder (ED) diagnoses (Bulik, Marcus, Zerwas, Levine, & La Via, 2012; Villarejo et al., 2012) may account for findings of a 70% prevalence of overweight and obesity in patients with BN or BED (Hudson, Hiripi, Pope, & Kessler, 2007; Kessler et al., 2013). Nonetheless, body composition, more than body weight categorization, seems to be important in evaluating the risk of physical health complications (Hamer, O'Donovan, Stensel, & Stamatakis, 2017). Evaluations of body composition need information, which has been largely missing in the ED literature, notably with respect to fat mass, regional body fat storage, lean body mass, visceral adipose tissue (VAT), and bone mineral density. Women with BN in the lower and upper BMI categories have been shown to have lower and higher body fat percentage (%BF), respectively, compared with healthy controls (Probst et al., 2004). Moreover, increased VAT is reported among weight‐restored females with anorexia nervosa (AN) (Iketani, Kiriike, Nagata, & Yamagami, 1999) as well as among women with BN or BED (Ludescher et al., 2009). Findings on bone mineral density in BN have been equivocal, whereas information on bone mineral density in women with BED is sparse (Robinson, Aldridge, Clark, Misra, & Micali, 2016; Solmi et al., 2016).

A history of AN may be decisive on findings of current body weight, body composition, and bone mineral density. Hence, when evaluating the interrelationship between BN or BED, body weight and composition, history of AN might be important to consider. A few studies addressing this interrelationship have shown that a history of AN is an important determinant of current body weight and variables of body composition in persons with current diagnosis of BN compared with those with no history of AN (Naessén, Carlström, Glant, Jacobsson, & Hirschberg, 2006; Robinson et al., 2016; Vaz, Guisado, & Peñas‐Lledó, 2003). Moreover, how different EDs relate to level of physical activity, physical fitness, and body composition is hampered by the fact that only a limited number of health variables are examined and with various methods. In particular, the wide use of subjective and self‐report measures is a consistent source of underestimation of CRF and physical activity among individuals with ED (Bratland‐Sanda et al., 2010a; Soundy, Taylor, Faulkner, & Rowlands, 2007).

The present study aims to provide extensive information about objectively measured physical fitness (physical activity level, blood pressure, CRF, muscle strength, body weight history and current body weight, body composition, and bone mineral density) among women with BN or BED and to describe the results relative to normative or recommended levels. We also compare individuals according to a history of AN, regardless of current ED diagnosis. We hypothesize that persons with BN or BED have impaired physical fitness as compared with normative or recommended values and that values for body composition and bone mineral density are lower in women with a history of AN compared with those with no history of AN.

## 2 METHOD

**Figure 1:**
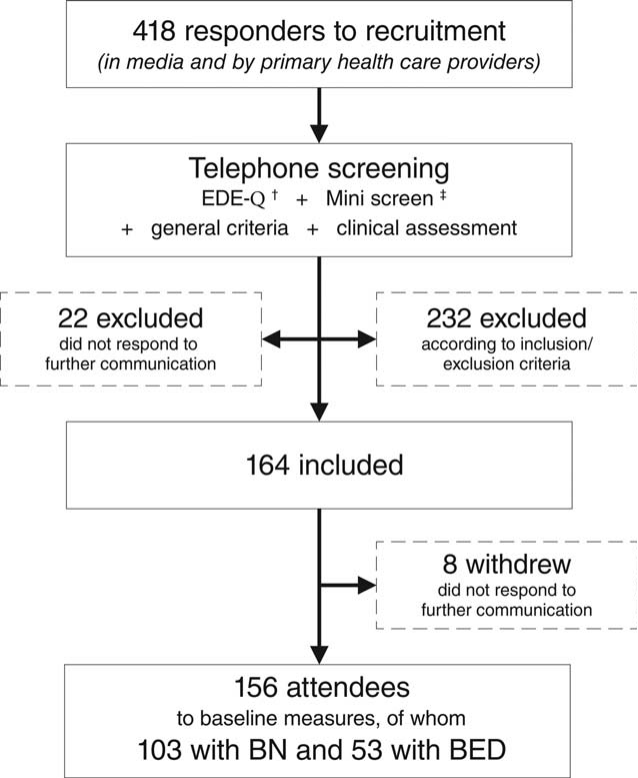
Flow chart of inclusion of participants to the present study based on who were recruited for participation in the original RCT studying the effects of cognitive behavior therapy (CBT) versus a physical exercise and dietary **t**herapy treatment program (PED‐t) (Mathisen et al., 2017). *Note*. ^†^Fairburn et al. (2008), ^‡^Sheehan et al. (1997)

For the purpose of the present study, 156 female participants were included. They represented the full eligible sample recruited for a randomized controlled trial to investigate whether a new physical exercise and dietary therapy treatment program may reduce symptoms of BN and BED equally to cognitive behavioral therapy (Mathisen et al., 2017). Figure 1 provides an overview of the recruitment and screening procedures. Responders to recruitment were considered for inclusion if they were between 18 and 40 years of age, had a BMI range between 17.5 and 35, a Diagnostic and Statistical Manual of Mental Disorders, Fifth Edition (DSM‐5) diagnosis of BN or BED with mild‐to‐severe symptoms. Diagnosis was based on information from Eating Disorder Examination Questionnaire 6.0 (EDE‐Q), self‐reported behavior according to DSM‐5 diagnostic criteria (printed form), and finally confirmed by clinical assessment. A final inclusion required signed confirmation from the participant and their general practitioner. Women who were currently pregnant, a competitive athlete, had a concurrent severe symptom or personality disorder in need of other treatment options, and those who had received cognitive behavior therapy for ED for the last two years before the study were excluded. All participants attending the assessment at baseline before the commencement of the mentioned treatment are included in the present descriptive study. The study has been approved by the Norwegian Regional Committee for Medical and Health Research Ethics (ID: 2013/1871) and registered in Clinical Trials (ID: NCT02079935).

### 2.1 Physical activity, CRF, and muscle strength

Sedentary time and physical activity were objectively measured for 7 consecutive days using the ActiGraph accelerometer (ActiGraph GT3x and GT3x+; Actigraph, LCC, Pensacola, FL) placed on their right hip. It was only removed for water activity and night‐time sleep. All accelerometers extract data from the vertical axis in 60‐s epochs with 30 Hz sampling rate. Nonwear time was determined as continuous zero‐count epochs lasting at least 60 min (allowing for two exceptions). Wear days were deemed valid if worn for at least 600 min/day and a minimum of two valid days. Intensity‐specific physical activities were derived using the Troiano cut‐points (Troiano et al., 2008), and continuous bouts of moderate‐to‐vigorous physical activity (MVPA) were determined by summing the time (in min) of at least moderate intensity that was a part of a bout of MVPA minutes lasting at least 10 min (allowing for two drops in intensity before terminating the bout).

All participants were instructed to fast and to travel using passive transportation to the laboratories at the Norwegian School of Sport Sciences from 7:30 to 10:00 a.m. Resting blood pressure was measured twice according to a standardized protocol (Mancia et al., 2013) with an automatic blood pressure device (Spot Vital Signs LXi; Welch Allyn, Skaneateles Falls, NY). The average recordings were used.

One to three hours after the participants completed the body composition measurement and ate breakfast, their CRF was measured by performing a cardiopulmonary exercise test on a treadmill (ELG 90/200 Sports; Woodway, Weil am Rhein, Germany) with an incremental modified Balke protocol until exhaustion (Edvardsen, Hem, & Anderssen, 2014). Gas exchange was measured using a breath‐by‐breath gas analysis system (OxyconPro analyzer; Jaeger, Würtzburg, Germany) with a Hans Rudolph two‐way breathing mask (2700 series; Hans Rudolph, Kansas City, KS). Measures of respiratory exchange ratio (RER) ≥ 1.10, and lactate concentration ≥ 7.0 mmol/L measured 1 min after test termination and analyzed immediately in a 1500‐Sport‐lactate analyzer (YSI, Yellow Springs Instruments, Yellow Springs, OH), were required to ensure a valid measure of maximal oxygen uptake (VO_2max_) (Edvardsen et al., 2013). A Borg scale rating ≥ 17 was additionally required to approve the test result (Borg, 1982; Haff & Dumke, 2012).

Maximal strength tests (one repetition maximum, 1RM) followed the cardiopulmonary exercise test in the following order: squats in machine, bench press, and seated cable row. These three tests were performed according to predefined performance criteria and initiated with standardized warm‐up sets of 10–8–6–4 repetitions.

### 2.2 Body composition and bone mineral density

Participants were weighed in their underwear, and their height was measured with a fixed stadiometer (Seca scale, Mod: 8777021094, S/N: 5877248124885, Seca Deutschland, Hamburg, Germany). A dual‐energy X‐ray absorptiometry (Lunar iDXA, enCORE Software, version 14.10.022; GE Healthcare, Madison, WI) performing a three‐site scan (lumbar area [L2‐L4]; femoral neck, trochanter, and shaft [proximal femur]; whole body) was used to measure body composition (fat mass, %BF, lean body mass, VAT, android‐to‐gynoid fat mass ratio [AG ratio], bone mineral content, and bone mineral density). All data were analyzed according to the guidelines (Nana, Slater, Stewart, & Burke, 2015). Recommended %BF for premenopausal females was set at <33%, based on an evaluation of previous studies (Imboden et al., 2017a; Okorodudu et al., 2010). By combining BMI and %BF information, we defined a woman as having “masked obesity” if BMI < 25 and %BF ≥ 33%.

### 2.3 Questionnaires and retrospective information

All participants completed the EDE‐Q (Fairburn, 2009) and provided information on ED‐history, body weight fluctuation, and menstrual history (Mathisen et al., 2017). The EDE‐Q cutoff scores 2.62 and 2.63 have been identified as valid in identifying BN and BED, respectively, among Norwegian adults (Rø, Reas, & Stedal, 2015).

### 2.4 Reference measures

For comparative reasons, we included the recommended or normative levels for physiological fitness in Section 3, all presented consecutively with corresponding references.

### 2.5 Statistical analyses

All data were analyzed with SPSS version 24 (IBM, Armonk, NY). The results were presented as mean (standard deviation [*SD*]) unless otherwise stated. For evaluation of the physiological variables mean values of norms and recommendations were added when attainable, and with reference to the more descriptive purpose of this study, a liberal 99% confidence interval was used to enable statistical comparisons.

Multiple linear regressions were calculated separately for BN and BED to identify models to predict %BF, VAT, and bone mineral density in the spine and proximal femur, respectively. Excluded from the models were variables that were statistically nonsignificant in the bivariate correlations.

Inspection of the scatter plot of VAT and %BF indicated a distinct bend in the curve at roughly the same location as previously reported in a piecewise regression model (i.e., at 38.8% BF) (Bosch et al., 2015). We therefore performed a similar analysis and searched empirically for the cutoff point that maximized *R* ^2^ (explained variance) in our study. The piecewise regression model was specified as $f\left(VAT\right)=b_{0}+b_{1}BF\%+b_{2}{BF\%}_{high}$. BF%_high_ was coded as BF% if BF%≥00 if BF%<0, 0 representing the centered BF% value around the chosen cutoff.

The results from participants who had errors in the registration of physical parameters, and from 30 participants in the presentation on history of body weight fluctuations (due to missing self‐reports), were excluded from the respective and relevant analysis.

Participants with a history of AN were compared with those with no such history by analysis of variance and Mann–Whitney *U* test. Due to multiple comparisons, a Bonferroni correction (*p* = .05/17 tests) was used to reduce the family‐wise error rate and *p* values <.003 were considered statistically significant. Standardized mean differences were calculated as Hedge's *g* (Hedge & Olkin, 1985). These effect sizes were interpreted as small, moderate, or large if larger than .2, .5, or .8, respectively. The results of 19 participants were excluded from the analysis because of missing information on history of AN.

## 3 RESULTS

The mean (*SD*) age and BMI for all included were 28.4 (5.7) years and 25.3 (4.8) kg/m^2^, respectively. The characteristics of included participants are presented in Table 1 according to diagnosis.

**Table 1:** Characteristics of participants with BN or BED related to age and illness history

variable	BN		BED	
	*n*	*M* (*SD*)	*n*	*M* (*SD*)
Age (years)	103	27.8 (5.5)	53	29.5 (6.1)
Duration of illness (years)	103	11.9 (6.6)	53	13.5 (8.1)
EDE‐Q, global score	102	3.8 (.9)	53	3.6 (.9)
Previous history of AN, *n* (%)	32 (36.0%)		5 (10.4%)	

### 3.1 Physical activity, CRF, and muscle strength

Less than half of the participants with BN or BED complied with recommendations for physical activity, whereas one third of the participants in each of the diagnostic groups had high levels of physical activity (i.e., ≥30 continuous minutes of MVPA) (Table 2). Increased blood pressure was found among 24.7% and 28.0% of participants with BN and BED, respectively, of whom seven (6.9%) participants with BN and three (6.0%) participants with BED were classified as hypertensive for either systolic or diastolic blood pressure. The mean CRF among participants with BED, and the CRF in 20% of participants with BN, was below the previously suggested healthy threshold of 35.1 mL kg^−1^ min^−1^ (Aspenes et al., 2011).

**Table 2:** Physical fitness in participants with BN or BED, and healthy normative (no) or recommended (re) levels

Physiologic variable	BN		BED		Normative (no)/recommended (re)
	*n*	*M* (*SD*) [CI]	*n*	*M* (*SD*) [CI]	
Physical activity level per day, counts per min	81	457.2 (163.2) [409.3, 505.0]	44	433.5 (153.4) [367.1, 493.9]	349.0 (141.0) (no)^a^
Average time spent in moderate to vigorous activity, min/day	81	24.4 (19.8) [18.6, 30.2]	44	23.0 (20.3) [15.3, 31.9]	15.0 (.8) (no)^a^/21.4 (re)^a^
Physically active according to recommendations, *n* (%)	81	38 (46.9%)	44	19 (43.2%)	28.5% (CI: 24.1, 32.9) (no)^a^
Average sedentary time per day (min/day)	81	601.2 (60.7) [583.4, 618.9]	44	599.8 (60.1) [574.7, 624.2]	547.0 (4.0) (no)^a^
Systolic blood pressure (mmHg)	101	118.3 (12.7) [115.0, 121.6]	50	121.9 (10.4) [118.0, 125.9]	120–129 (re)^b^
High normal systolic blood pressure, *n* (%)	13 (12.9%)		9 (18.0%)		130–139 (re)^b^
Diastolic blood pressure (mmHg)	101	75.6 (7.6) [73.6, 77.6]	50	78.4 (6.9) [75.8, 81.1]	80–84 (re)^b^
High normal diastolic blood pressure, *n* (%)	6 (5.9%)		7 (14.0%)		85–89 (re)^b^
Maximal oxygen uptake (L min^−1^)	100	2.65 (.45) [2.53, 2.76]	52	2.73 (.49) [2.54, 2.91]	2.60 (.44) (no)^c^
Maximal oxygen uptake (mL BW^−1^ min^−1^)	100	40.7 (6.8) [38.9, 42.5]	52	34.6 (8.1) [31.6, 37.6]	40.0 (7.3) (no)^c^
Maximal oxygen uptake (mL LBM^−1^ min^−1^)	100	60.5 (7.7) [58.4, 62.5]	52	58.5 (8.9) [55.2, 61.8]	54.7 (8.6) (no)^c^
Squat, 1RM (kg)	96	65.4 (19.2)	46	61.4 (17.6)	
Squat, relative 1RM (kg BW^−1^)	96	1.00 (.31)	46	.79 (.25)	
Bench press, 1RM (kg)	96	37.8 (10.1)	49	37.6 (9.5)	
Bench press, relative 1RM (kg BW^−1^)	96	.58 (0.16) [.54, .62]	49	.47 (.13) [.42, .53]	.39 (.08) (no)^d^
Seated row, 1RM (kg)	96	33.6 (8.2)	46	34.6 (5.9)	
Seated row, relative 1RM (kg BW^−1^)	96	.51 (.13)	46	.44 (.09)	

*Note*. LBM = lean body mass; 1RM = one repetition maximum. 99% Confidence intervals (CI) are presented for variables with recommended or normative values, for comparisons.

a Hansen et al. (2015).

b Mancia et al. (2013).

c Edvardsen et al. (2013) (mean values of age group 20–29 and 30–39).

d Brown et al. (1998) (mean values of age group 20–29 and 30–39).

### 3.2 Body composition and bone mineral density

The majority of participants with BN had normal weight, whereas the majority of participants with BED were overweight or obese (Table 3). Moreover, 12 (12%) participants with BN and 5 (9.4%) participants with BED had masked obesity.

**Table 3:** Body mass index (BMI) and body fat percentage (%BF) in participants with BN or BED

	BN (*n = 103*)		BED (*n = 53*)	
BMI class	% Of participants	%BF M (*SD*)	% Of participants	%BF M (*SD*)
Underweight (BMI < 18.5)	5.8	18.7 (2.5)	—	—
Normal weight (BMI 18.5–24.9)	61.2	27.4 (6.2)	28.3	31.2 (5.5)
Overweight (BMI 25–29.9)	28.2	36.2 (6.0)	30.2	37.1 (7.0)
Obese (BMI ≥ 30)	4.9	44.5 (4.4)	41.5	46.9 (4.4)
%BF ≥ 33%, *n* (%)	39 (37.9%)		40 (75.5%)	

The mean BF% and mean VAT were below recommended upper thresholds in participants with BN, but above such thresholds in participants with BED (Table 4). Overall, the AG ratio correlated well with VAT in both samples. However, a small variation in VAT was observed with AG ratio < 1.0, whereas there was a strong, correlational increase in VAT with AG ratio > 1.0.

**Table 4:** Anthropometrics and body composition in participants with BN or BED, and normative (no) or recommended (re) levels

Soft body tissue variables	BN		BED		Normative (no)/Recommended (re)
	*n*	*M* (*SD*) [CI]	*n*	*M* (*SD*) [CI]	
Body weight (kg)	103	66.2 (11.8)	53	81.4 (15.5)	
Height (cm)	103	167.6 (6.5)	53	168.1 (6.7)	
BMI (kg m^−2^)	103	23.5 (3.6) [22.6, 24.4]	53	28.8 (5.1) [26.9, 30.7]	18.5–24.9 (re)^a^
Total adult BW difference^b^ (kg)	86	23.4 (12.8)	40	33.4 (21.3)	
Lean body mass (kg)	102	43.8 (5.4) [42.5, 45.3]	53	45.8 (8.5) [44.4, 48.8]	43.3 (6.45) (no)^e^
Fat mass (kg)	102	19.9 (8.5)	53	32.3 (12.3)	
Body fat percent (%)	102	30.2 (8.2) [28.1, 32.3]	53	39.5 (8.6) [36.3, 42.7]	<33% (re)^d^ 34% (9.75) (no)^f^
VAT (g)	102	225.8 (293.9) [149.4, 302.2]	53	606.2 (545.5) [405.4, 806.2]	300.0 (300.0) (no)^c^
AG ratio	102	.73 (.24) [.67, .79]	53	.92 (.20) [.85, .99]	.36 (.14)^d^

*Note*. BW = body weight; VAT = visceral adipose tissue; AG‐ratio = ratio of android‐to‐gynoid fat tissue percentage. 99% Confidence intervals (CI) are presented for variables with recommended or normative values, for comparisons.

a World Health Organization (1995).

b Adult total body weight difference is the difference between the lowest and the highest body weight after 18 years of age.

c Bosch et al. (2015), normative values based on interpretation of results.

d Imboden et al. (2017a), mean values of age group 20–29 and 30–39.

e Imboden et al. (2017b), mean values of age group 20–29 and 30–39.

f Okorodudu et al. (2010).

Among participants with BN, BMI (β = 1.76, 95% confidence interval, CI, [1.46, 2.07]) and MVPA (in 10‐min increments) (β = −.75, 95% CI [−1.21, −0.30]) accounted for 65.9% of the %BF variation (*F*[2,78] = 78.19, *p <*.001). With respect to BED, BMI alone accounted for 67.2% of the variation in %BF (β = 1.39, 95% CI [1.12, 1.66]) (*F*[1,51] =107.6, *p* <.001).

For participants with BN, BMI alone accounted for 51.2% of the variation in VAT (β = 59.72, 95% CI [48.26, 71.19]) (*F*[1,100] = 106.8, *p* <.001). By contrast, 68.2% of the variation in VAT among participants with BED was accounted for by BMI (β = 80.24, 95% CI [63.23, 97.26]) and CRF (β = −0.34, 95% CI [−0.51, −0.16]) (*F*[3,49] =55.7, *p* < .001).

**Figure 2:**
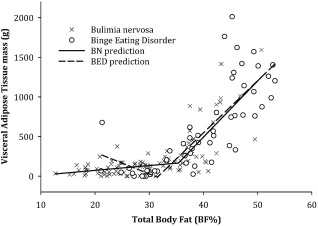
The non‐linear relationship between visceral adipose tissue (VAT, g) and percentage total body fat (BF%) according to a piecewise regression model with separate slopes and BF% cutoff levels for bulimia nervosa and binge‐eating disorder, respectively

The piecewise regression models with %BF as the predictor and VAT as the outcome showed a maximum *R* ^2^ using BF% cutoff values of 35.7% and 31.9% BF for BN and BED, respectively (Figure 2). The first linear part predicted 44.3% and 56.4% of variation, respectively, whereas the additional modeling of the steeper increase in VAT after the cutoff points predicted an additional 19.6% and 8.1%, respectively (*p* < .001 for all). For BN, the β coefficients were 6.1 (*p* = .07) and 64.9 (*p* <.001), thus yielding an increase of 71 g VAT per unit change in BF% after the BF% cutoff point (35.7%). For BED, the β coefficients were −27.3 (*p* = .24) and 93.1 (*p* < .001), thus yielding an increase of 65.8 g VAT per unit change in BF% after the BF% cutoff point (31.9%).

Three (2.9%) participants with BN and six (11.3%) participants with BED had spine Z‐scores corresponding to low bone mineral density, and one participant with BN had a spine T‐score corresponding to a diagnosis of osteoporosis. Furthermore, one participant with BN and one participant with BED had proximal femur Z‐scores corresponding to low bone mineral density. Results for bone mineral density are presented in Table 5.

**Table 5:** Bone mineral content and density in participants with BN or BED, with recommended *Z*‐scores for comparisons

Bone health variables	BN		BED		Recommended
	*n*	*M* (*SD*) [CI]	*n*	*M* (*SD*) [CI]	
Total bone mineral content (g)	102	2483.3 (331.2)	53	2581.3 (314.5)	
Total bone mineral density (g cm^−2^)	102	1.197 (0.109)	53	1.236 (0.086)	
Total bone mineral density, *Z*‐score	102	1.1 (.9) [.87, 1.34]	53	0.9 (.9) [.59, 1.21]	>–2.0^a^
Bone mineral density spine, (g cm^−2^)	102	1.182 (0.148)	53	1.215 (0.113)	
Bone mineral density spine, *Z*‐score	102	−0.2 (1.1) [–.42, .13]	53	−0.3 (1.0) [–.71, .03]	>–2.0^a^
Bone mineral density femur (g cm^−2^)	102	1.020 (0.131)	53	1.044 (0.105)	
Bone mineral density femur, *Z*‐score	102	.14 (.94) [–.11, .38]	53	.03 (.84) [–.28, .34]	>–2.0^a^

*Note*. ^a^ISCD (2015), World Health Organization (2004). For comparison, 99% confidence intervals (CI) are presented for variables with recommended or normative mean values available.

Among participants with BN, BMI (β = 0.015, 95% CI [0.007, 0.023]) and lean body mass (β = 0.007, 95% CI [0.002, 0.012]) accounted for 28.8% of spine bone mineral density variations [*F*(2,99) = 21.4, *p* < .001]. No significant determinants were detected for participants with BED.

For BN, BMI (β = .02, 95% CI [.02, .03]) and 1RM squat (β = .002, 95% CI [.001, .003]) accounted for 45.0% of variance in proximal femur bone mineral density [*F*(2,93) =  39.8, *p* < .001]. No variables were related to femur bone mineral density among participants with BED.

### 3.3 History of AN

Overall, participants with a history of AN had lower values on variables related to body composition compared with those with no history of AN, with effect size >.6 (Table 6).

**Table 6:** Characteristics for participants with or without a history of anorexia nervosa (AN) (irrespective of current diagnosis)

Characteristics and body composition variables	Previous AN		No previous AN		*p*	Hedge's *g*
	*N*	*M* (*SD*)	*n*	*M* (*SD*)		
Age for illness onset (years)	37	15.2 (3.1)	100	16.3 (4.6)	.28	–.26
Duration of illness (years)	37	12.0 (6.5)	100	13.0 (7.6)	.49	–.14
Total adult BW difference^a^ (kg)	28	21.7 (8.0)	80	28.2 (16.3)	.17	–.44
Adult lowest BW (kg)	31	49.7 (9.9)	88	55.9 (10.9)	.005	–.58
Adult highest BW (kg)	30	71.0 (9.9)	83	83.5 (17.9)	.001	–.77
Current BW (kg)	37	62.2 (10.4)	100	75.1 (14.7)	.000	–.94
Height (cm)	37	167.5 (5.9)	100	167.7 (6.5)	.91	–.03
BMI (kg m^−2^)	37	22.1 (3.4)	100	26.7 (4.7)	.000	−1.05
Physical activity level per day (counts per min)	27	465.5 (176.8)	87	424.5 (166.9)	.08	.24
Maximal oxygen uptake (mL min^−1^)	36	2586.5 (540.7)	97	2731.8 (427.8)	.11	–.32
Maximal oxygen uptake (mL BW^−1^ min^−1^)	36	42.0 (8.1)	97	37.5 (7.4)	.005	.59
Fat mass (kg)	36	17.4 (8.3)	100	26.8 (11.4)	.000	–.88
Lean body mass (kg)	36	42.4 (5.4)	100	45.6 (5.5)	.003	–.58
Body fat percent (%)	36	28.0 (9.1)	100	35.7 (8.9)	.000	–.86
VAT (g)	36	173.8 (219.5)	100	441.9 (465.3)	.002	–.65
Total bone mineral density (g cm^−2^)	36	1.163 (0.106)	100	1.226 (0.943)	.001	–.63
Bone mineral density spine (g cm^−2^)	36	1.136 (0.141)	100	1.216 (0.130)	.002	–.60
Bone mineral density femur, (g cm^−2^)	36	0.986 (0.125)	100	1.045 (0.118)	.01	–.49
Current diagnosis of BN, *n* (%)^b^	32 (86.5%)		57 (57%)		—	
Current diagnosis of BED, *n* (%)^b^	5 (13.5%)		43 (43%)		—	

*Note*. BN = bulimia nervosa; BED = binge‐eating disorder; BW = body weight; BMI = body mass index; BMD = bone mineral density; VAT = visceral adipose tissue.

a Adult total BW difference is the difference between the lowest and the highest BW after 18 years of age.

b Number with BN or BED significant different between those with and without history of AN, *p* = .001.

## 4 DISCUSSION

Overall, results from this study showed that women with BN or BED were more physically active than a national healthy cohort of a comparable age span and sex, still less than half of the participants with BN or BED met the official minimal recommendations for physical activity. Furthermore, women with BN were comparable with the healthy population in physical fitness, whereas women with BED scored lower on maximal oxygen uptake and higher on BMI, BF%, and VAT than the normative or recommended levels. Despite overall average normal findings on physical fitness, we identified high numbers of women with BN or BED with unfavorable CRF and body composition and up to 12% had masked obesity. Furthermore, after comparing participants with history of AN to those with no such history, we found that the former had generally lower values on physical fitness variables.

### 4.1 Physical activity, CRF, and muscle strength

The finding of a higher level of physical activity and time spent in MVPA in both diagnostic groups, compared with a national cohort study (Hansen et al., 2015), replicated a previous finding, being the first to report on physical activity in patients with ED using objective measures (Bratland‐Sanda et al., 2010a). The finding from the current study was mainly because of higher volume of continuous time spent in MVPA per day. Nevertheless, the recommendation relates to a minimum of physical activity. The fact that less than half of the participants in this study complied with the recommended weekly MVPA (Haskell et al., 2007), and spent about 10 hr being sedentary, makes it fair to hypothesize that such a sedentary lifestyle among women with BN and BED increases the risk of medical comorbidity (Myers et al., 2015; Warburton, Nicol, & Bredin, 2006).

The mean blood pressure values were within the normal range (Mancia et al., 2013), but about one third of participants with BN and BED, respectively, had increased blood pressure or hypertension, indicating an increased risk of cardiovascular disease (Mancia et al., 2013).

The mean CRF scores (VO_2max_) for participants with BN were comparable to normative values (Edvardsen et al., 2013), but approximately 20% scored below the previous identified healthy threshold (Aspenes et al., 2011), and for the BED group, the mean score was below this threshold. Previous studies have found CRF to be a more important predictor for mortality than any other lifestyle factor (Ross et al., 2016). The low VO_2max_ in participants with BED raises concern about the long‐term health risk in this specific subsample.

Normative values for muscular strength are only available for one of the tested exercises. Both groups in the present study were above such normative values (Brown & Miller, 1998), probably because very few were underweight and at risk for atrophy and myopathy, often found in patients with AN (McLoughlin et al., 1998; Nicholls, Wells, Singhal, & Stanhope, 2002).

Our findings on high numbers of participants not complying with the minimal recommendation for physical activity, having high blood pressure, and a low CRF among participants with BN or BED raise concern about their morbidity.

### 4.2 Body composition and bone mineral density

The mean %BF, when sorted by BMI categories in participants with BN, was comparable with a previous report (Probst et al., 2004). Despite the mean BMI and %BF being within a healthy range, a large proportion of the participants with BN or BED was overweight or obese. BMI served as a good indicator of body composition in our sample. However, the fact that 12% (BN) and 9.4% (BED) had masked obesity replicated previous findings. Here, a BMI ≥ 30 corresponded well with morbid body composition, but a BMI < 30 did not always correspond well with %BF or cardiometabolic health (Bratland‐Sanda, Martinsen, & Sundgot‐Borgen, 2012; Swainson, Batterham, Tsakirides, Rutherford, & Hind, 2017; Tomiyama, Hunger, Nguyen‐Cuu, & Wells, 2016). Hence, the customary practice of using BMI as a health indicator for individuals with ED may be questioned.

VAT is associated with a range of risk factors for lifestyle‐related diseases, such as insulin sensitivity and triglyceride and cholesterol levels (Bi et al., 2015; Rothney et al., 2013; Sasai et al., 2015). Eighteen (17.5%) participants with BN and 33 (62.3%) participants with BED had VAT levels above a previous suggested normative, healthy level of 300 g (Bosch et al., 2015). In this respect, BMI was the most important determinant for VAT in participants with BN, accounting for 51.2% of the variation, and with the addition of CRF for participants with BED, 68.2% of variation in VAT was accounted for. Previous studies suggest an unfavorable metabolic profile with increased VAT levels and with low VO_2max_, separately (Aspenes et al., 2011; Ross et al., 2016). In our multiple linear regression models for VAT levels, CRF was negatively related to VAT in participants with BED. Moreover, low CRF has previously been associated with high VAT accumulation in men (Arsenault et al., 2007), and we found such associations among 50% of those having BN and low VO_2max_, and among all but two of those with BED and low VO_2max_.

We found a sudden bend in the curve between VAT and %BF when assessed by exploratory observation. A previous study reported a bend at 38.8%BF using a piecewise regression model (Bosch et al., 2015). We found cutoff areas for the relationship between VAT and %BF to be close to this previously identified threshold in both participants with BN and with BED (35.7% and 31.9%, respectively). Noticeably, these cutoffs were both close to the %BF we evaluated as the upper threshold for a healthy body composition (i.e., 33%), thus supplementing the literature of body composition on evaluation of a healthy %BF.

The waist‐to‐hip ratio, or the AG ratio, is used as indication of VAT accumulation. In our sample, an AG ratio <1.0 presented with small variation in VAT, but with a strong, correlational increase in VAT when >1.0. This might imply that the mean AG ratios observed in our sample were within a healthy range, even when being above a previously suggested normative level (Imboden et al., 2017a). Moreover, among the participants with BN and BED, 15% and 42%, respectively, had an AG ratio >1, and all but two of these had VAT levels above normative levels. Still, about one third of those with high VAT was not identified by an AG ratio >1.0. This implies that the AG ratio, or an extension to this, the use of waist‐to‐hip ratio, is not successful at identifying all at risk for metabolic impairment observed with high VAT levels.

Surprisingly, low bone mineral density seemed to be more frequent in participants with BED compared with those with BN. The literature is unclear on the role of BED on bone mineral density; however, high body weight and low restrictiveness in eating behavior are assumed to protect bone mass (Goebel, Schweiger, Krüger, Fichter, 1999). Additionally, we found fewer participants with history of AN among those with BED compared with those with BN, a condition known to cause low bone mineral density (Robinson et al., 2016; Singhal et al., 2018). However, irrespective of diagnosis, low scores in spine BMD were the most prevalent site‐specific finding, replicating previous studies on bone mineral density among individuals with BN (Naessén et al., 2006; Newton, Freeman, Hannan, & Cowen, 1993; Robinson et al., 2016; Solmi et al., 2016). For participants with BN, variables related to physical fitness (lean body mass and squat strength), together with BMI, were found to account for variations in bone mineral density. In contrast to previous findings (Robinson et al., 2016), history of AN has no effect on bone mineral density, yet the importance of our finding is difficult to evaluate because of the lack of information about the onset or the duration of AN.

Even if BMI proved an important variable in most of our multiple linear regression analysis, it did not successfully identify all with impaired fitness and masked up to 12% with morbid body composition. The results of our analysis further highlighted the role of CRF and muscle strength for optimal body composition (i.e., to constrain %BF and VAT levels and improve bone mineral density).

### 4.3 History of AN

We found differences in body composition when evaluating the participants according to history of AN and irrespective of current diagnosis. Hence, our findings support previous suggestion that those with a history of AN seem to preserve some of the restrictive behavior after remission or transition into other ED diagnosis (Vaz et al., 2003).

### 4.4 Limitations

The use of participants recruited from the general population increases the generalizability, as studies based on treatment‐seeking patients or in‐treatment populations only reflect the minor proportion that ever seek help. Furthermore, the use of objectively based high‐technology devices for physical health and activity measures increases the credibility and validity of the findings.

The present findings may not apply to markedly obese women with BN or BED because the participants were recruited from an RCT using BMI >35 as an exclusion criterion. In addition, the findings may not necessarily apply to males. This is important considering the sex distribution being more even with BED diagnosis (Hudson et al., 2007). Furthermore, a lack of objective information about weight history and a history of various ED diagnoses, notably AN, may raise a risk of underreporting, but it is expected to be negligible. Measuring VAT by magnetic resonance imaging and computed tomography would have yielded even more accurate data than the use of DXA, but high intercorrelations across these methods (Mohammad et al., 2017; Neeland, Grundy, Li, Adams‐Huet, & Vega, 2016; Reinhardt, Piaggi, DeMers, Trinidad, & Krakoff, 2017) indicate differences of negligible clinical significance (Neeland et al., 2016). Additionally, inclusion of an age‐ and BMI‐matched healthy control group would strengthen the conclusions about eating disorders and physical fitness as measured in the current study. However, as the intention of this article was to describe the physical fitness status of those with BN or BED approaching treatment, we consider the comparisons to normative or recommended levels as a sufficient first step.

## 5 CONCLUSIONS AND CLINICAL IMPLICATIONS

Overall, the women with BN or BED displayed adequate physical fitness. Nevertheless, the need for clinical attention to increase physical activity and CRF was justified, as a proportion of these women, notably those with BED, had impaired physical fitness and complied poorly with the recommendation for physical activity. Evaluation of physical fitness with the traditional BMI or waist‐to‐hip ratio (AG ratio in our work) did not well identify all with impaired physical fitness and left a high proportion with unidentified high VAT and up to 12% with masked obesity. Furthermore, CRF, lean body mass, muscle strength, and time in MVPA were all central determinants of VAT and %BF. These findings call for inclusion of physical fitness in routine clinical examinations and the need to integrate rather than restrict guided and facilitated physical activity and dietary recommendations in the treatment of women with BN or BED. Furthermore, with reference to the high correlation of CRF and physical fitness (Myers et al., 2015; Ross et al., 2016), and findings of improved correlation between regional fat storage and physical health, rather than BMI and physical helath, (Hamer et al., 2017), our results suggest that cardiopulmonary exercise testing and DXA can be ideally included in routine examination and screening of persons with EDs.

## CONFLICT OF INTEREST

The authors indicate no conflict of interest.
